# The Promise of DNA Methylation in Understanding Multigenerational Factors in Autism Spectrum Disorders

**DOI:** 10.3389/fgene.2022.831221

**Published:** 2022-02-15

**Authors:** Julia S. Mouat, Janine M. LaSalle

**Affiliations:** ^1^ LaSalle Laboratory, Department of Medical Microbiology and Immunology, University of California, Davis, Davis, CA, United States; ^2^ Perinatal Origins of Disparities Center, University of California, Davis, Davis, CA, United States; ^3^ MIND Institute, School of Medicine, University of California, Davis, Davis, CA, United States; ^4^ Genome Center, University of California, Davis, Davis, CA, United States

**Keywords:** autism spectrum disorder, DNA methylation, multigenerational, transgenerational, epigenetics, metabolism, neurodevelopment, precision medicine

## Abstract

Autism spectrum disorder (ASD) is a group of neurodevelopmental disorders characterized by impairments in social reciprocity and communication, restrictive interests, and repetitive behaviors. Most cases of ASD arise from a confluence of genetic susceptibility and environmental risk factors, whose interactions can be studied through epigenetic mechanisms such as DNA methylation. While various parental factors are known to increase risk for ASD, several studies have indicated that grandparental and great-grandparental factors may also contribute. In animal studies, gestational exposure to certain environmental factors, such as insecticides, medications, and social stress, increases risk for altered behavioral phenotypes in multiple subsequent generations. Changes in DNA methylation, gene expression, and chromatin accessibility often accompany these altered behavioral phenotypes, with changes often appearing in genes that are important for neurodevelopment or have been previously implicated in ASD. One hypothesized mechanism for these phenotypic and methylation changes includes the transmission of DNA methylation marks at individual chromosomal loci from parent to offspring and beyond, called multigenerational epigenetic inheritance. Alternatively, intermediate metabolic phenotypes in the parental generation may confer risk from the original grandparental exposure to risk for ASD in grandchildren, mediated by DNA methylation. While hypothesized mechanisms require further research, the potential for multigenerational epigenetics assessments of ASD risk has implications for precision medicine as the field attempts to address the variable etiology and clinical signs of ASD by incorporating genetic, environmental, and lifestyle factors. In this review, we discuss the promise of multigenerational DNA methylation investigations in understanding the complex etiology of ASD.

## 1 Introduction

Autism spectrum disorder (ASD) is a group of neurodevelopmental disorders that are characterized by deficits in social reciprocity and communication, restricted interests, and repetitive behavior. The prevalence of ASD has been steadily increasing over the past several decades, now affecting approximately one in 54 children in the United States (Maenner 2020). Most cases of ASD arise from a confluence of genetic predisposition, environmental factors, and gene-environment interactions (Bourgeron 2015), with the latter two potentially contributing to the apparent increase in prevalence in recent decades (Grether et al., 2009; King and Bearman 2009).

The Developmental Origins of Health and disease (DOHaD) hypothesis posits that environmental insults experienced during early development may impact the health of individuals later in life, as well as future generations. This framework fits the epidemiological and experimental findings that a wide range of environmental factors experienced *in utero* can affect risk for ASD. Prenatal risk factors with particularly strong evidence include advanced parental age, maternal medication use, and gestational diabetes (reviewed in Gardener et al., 2009; Karimi et al., 2017; Modabbernia et al., 2017), while folic acid may function as a protective factor (Levine et al., 2018). Due to the unequal exposure to risk factors and access to protective factors, this may generate an undue burden on underserved populations.

Further, the findings of several studies have indicated that certain environmental exposures may impact the health of individuals for multiple generations following the initial insult (reviewed in Breton et al., 2021). Human and animal studies are beginning to examine multigenerational risk factors for ASD, defined here as any factors that are non-genetic but may include demographic/social factors, chemical exposures, medications, and medical conditions in the generations prior to the individual diagnosed with ASD. The study of these potential multigenerational factors is warranted given the increase in ASD prevalence over the past several decades, which predominantly affects grandchildren of a population exposed to a surge of unregulated chemical pollutants in the mid 20th century (reviewed in Naidu et al., 2021).

Additionally, the field is working to identify potential mechanisms for transmission of risk through multiple generations. One of the most promising potential mechanisms is DNA methylation, an epigenetic mark that is hypothesized to be a mechanistic link between genetic and environmental risk (Law and Holland 2019), as well as a potential biomarker for ASD (Kimura et al., 2019; Zhu et al., 2019; Mordaunt et al., 2020; García-Ortiz et al., 2021; Garrido et al., 2021). Relatively few studies have examined DNA methylation in conjunction with grandparental or great-grandparental factors, despite the promising findings of existing multigenerational studies. This review describes the evidence for multigenerational risk for ASD in humans and animal models and explores the potential of DNA methylation for monitoring and/or counteracting multigenerational risk.

### 1.1 Autism Spectrum Disorder

ASD has no known single cause but numerous genetic and environmental factors that may individually contribute risk, and/or interact with one another. Estimates of heritability range widely, from 38% (Hallmayer et al., 2011) to 50% (Sandin et al., 2014), and upwards to 91% (meta-analysis by Tick et al., 2016). Most inherited genetic risk is predicted to arise from common variants (Gaugler et al., 2014) that have small effect sizes but are predicted to act in combination and have been associated with other outcomes such as schizophrenia, depression, and educational attainment (Grove et al., 2019). Rare *de novo* copy number and single nucleotide variants have been identified from exome sequencing of ASD and parent trios; these also overlap with variants associated with schizophrenia and intellectual disability (Iossifov et al., 2014). The genetic landscape of ASD has proven to be complex with hundreds of variants that may contribute to risk; however, to date no single gene or group of genes can predict or diagnose all cases of ASD.

Though ASD research has historically focused on genetic contributions to risk, newer studies have increasingly investigated environmental factors that may affect risk for ASD (reviewed in Bölte et al., 2019; Yoon et al., 2020). In line with the DOHaD hypothesis, studies have largely focused on prenatal and maternal factors, including demographics (e.g. parental age) (Reichenberg et al., 2006; King et al., 2009; Puleo et al., 2012); meta-analysis by Sandin et al. (2012), Taylor et al. (2019), maternal metabolic health (e.g. obesity and diabetes) (Li et al., 2016; Wang et al., 2019), chemical exposures (e.g. pesticides, air pollution, cigarette smoke) (Mitchell et al., 2012; meta-analysis by Jung et al., 2017; Granillo et al., 2019; meta-analysis by Chun et al., 2020), obstetric events (e.g. hypoxia) (Mann et al., 2010; Walker et al., 2015), and maternal medications (e.g. valproic acid) (Wiggs et al., 2020). Additionally, potential protective factors for ASD have been identified, including maternal folic acid (Levine et al., 2018) and dietary fat intake (Lyall et al., 2013). Prenatal and maternal factors have been thoroughly reviewed in previous publications (Gardener et al., 2009; Lyall et al., 2014; Wang et al., 2017; Hisle-Gorman et al., 2018; Katz et al., 2021).

### 1.2 DNA Methylation

The DOHaD hypothesis posits that the effects of early environmental exposures on health later in life may be mediated by epigenetic marks such as DNA methylation, which refers to the addition of a methyl group to carbon five on the pyrimidine ring of cytosine, forming 5-methylctyosine (5mC). In humans, DNA methylation occurs predominantly at CpG sites where the cytosine nucleotide on both strands is methylated, forming a symmetrical pattern. Approximately 70–80% of all cytosines in CpG nucleotides in the human genome are methylated (Kundaje et al., 2015), though this varies by cell type and location in the genome. Following development, global methylation levels remain nearly constant throughout the lifetime, though individual CpGs may change methylation status during further cellular differentiation (reviewed in Suelves et al., 2016), in response to extracellular signals (reviewed in Moore, Le, and Fan 2013), or in response to rhythmic cycles of diurnal metabolism (reviewed in Powell and LaSalle 2015). In contrast to gene expression which reflects the current state of cells, DNA methylation may reflect gene expression in other tissues (Gunasekara et al., 2019), *in utero* experiences, and past historical exposures of prior generations.

Multiple recent studies have identified a clear DNA methylation signature that can distinguish ASD from control DNA samples in multiple tissues, including sperm (Garrido et al., 2021), adult blood (Kimura et al., 2019), pediatric blood (García-Ortiz et al., 2021), umbilical cord blood (Mordaunt et al., 2020), and placenta (Zhu et al., 2019). While these studies fall short of being able to accurately predict ASD from differential methylation patterns, they do identify convergent genes such as *MECP2* (Nagarajan et al., 2006; Liyanage et al., 2019; Lu et al., 2020) and *GABRB3* (Wong et al., 2014). Research has yet to elucidate the mechanistic relationship between DNA methylation and ASD etiology or clinical variability but has shown a clear correlation between ASD diagnosis and altered patterns of DNA methylation across the genome.

## 2 Multigenerational Risk for ASD

**FIGURE 1:**
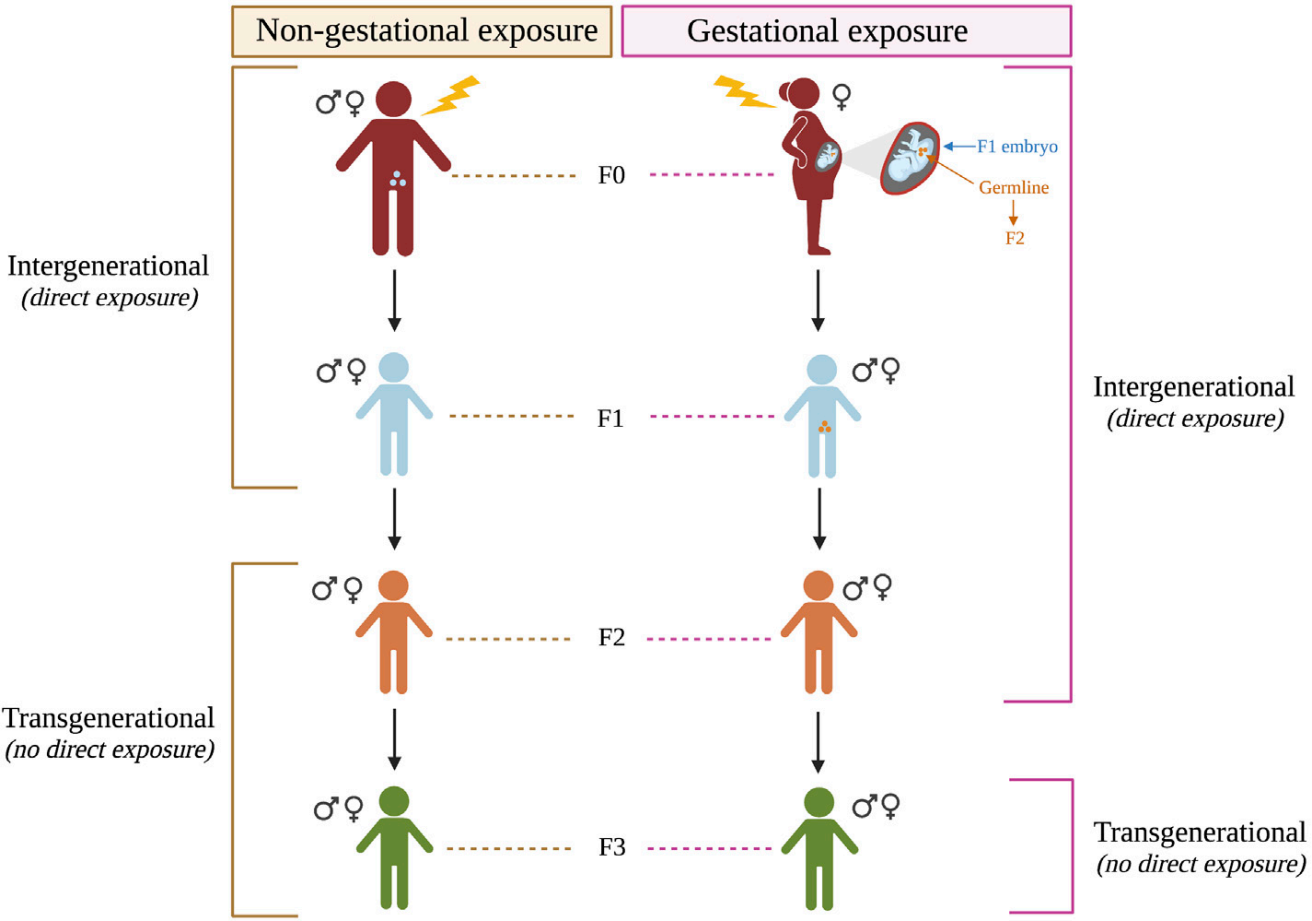
Multigenerational effects following gestational and non-gestational exposures. Multigenerational includes intergenerational effects from direct exposure to an environmental result and transgenerational effects from indirect exposure (i.e. the generations beyond the directly-exposed generations).

In addition to the prenatal and maternal factors discussed above, recent human and animal studies have shown support for multigenerational risk for ASD. In this review, the term ‘multigenerational’ encompasses ‘intergenerational’ factors, which affect generations that are directly exposed to an environmental insult (whether as an adult, fetus, or gamete), and ‘transgenerational’ factors, which affect later generations who were not directly exposed (Figure 1) (reviewed in Heard and Martienssen 2014; Tuscher and Day 2019). If the directly exposed individual (F0) is not pregnant (non-gestational exposure), then the F1 generation has exposure from the gametes, so the F2 is the first generation not directly exposed. However, if the exposure occurs during pregnancy (gestational exposure) the first non-exposed generation is F3 since the fetus (F1) and their gametes (F2) are directly exposed to the insult. For a molecular mechanism to be transgenerational, it must produce effects in generations that were not directly exposed. While human studies have been primarily limited to intergenerational factors, several animal studies have examined potential transgenerational factors, as discussed in the next sections.

### 2.1 Intergenerational Risk Factors

Below is a discussion of potential risk factors for ASD that have been identified from three-generation human studies, with animal studies often supporting the findings. Behavioral changes in animals are used as a proxy for neurodevelopmental diagnoses in humans, though the measured phenotypes range across studies. Major categories of identified intergenerational risk factors are advanced age, medication use, chemical exposure, and social stress; both maternal and paternal lineages are implicated, though each shows distinct patterns.

#### 2.1.1 Age

Advanced parental and grandparental age, particularly in the paternal line, has been one of the factors most strongly associated with ASD. For example, risk for ASD is increased if the individual’s grandfather fathered children at an advanced age (Frans et al., 2013; Gao et al., 2020). This finding is independent of the parent’s age at the grandchild’s birth, suggesting that risk for ASD may develop over generations. In mice, advanced age of the F0 male during breeding led to increased ultrasound vocalization activity, decreased sociability, increased grooming activity, and increased anxiety-like responses in both the F1 and F2 generations (Sampino et al., 2014).

#### 2.1.2 Medication Use

Certain medications, particularly when taken during pregnancy, may increase risk for ASD. For example, maternal grandmother use of diethylstylbestrol (DES), a synthetic form of estrogen, during pregnancy is associated with an increased risk of ADHD in male and female grandchildren (Kioumourtzoglou et al., 2018). A case-study of one family found that children and grandchildren from DES-free pregnancies did not develop psychiatric disorders, while those from DES-exposed pregnancies (from the same mother/grandmother) were highly likely to develop psychiatric disorders, including ASD (Soyer-Gobillard et al., 2021).

#### 2.1.3 Chemical Exposure

Chemical exposures can range widely, from cigarette smoke to pesticides, and many may have effects on neurodevelopment (reviewed in Fujiwara et al., 2016). For instance, maternal grandmother smoking is associated with granddaughters having adverse scores in Social Communication and Repetitive Behavior measures, which are predictive phenotypes of ASD (Golding et al., 2017). Interestingly, this finding holds true only if the mother did not smoke during pregnancy. In mice, nicotine exposure induced attention-deficit/hyperactivity disorder (ADHD)-like behavior such as greater hyperactivity and risk-taking behavior in both the F1 and F2 generations (Buck et al., 2019). ADHD is a common co-occurrence of ASD (van der Meer et al., 2012; Russell et al., 2014) and the two diagnoses share overlapping phenotypes (Ronald et al., 2014).

Several insecticides have been identified as potential risk factors for ASD; permethrin, a widely used insecticide and known carcinogen, has been shown to impact the behavior of several generations following exposure. Exposure of F0 zebrafish to permethrin during early development through 28 days post-fertilization affects F0 fertility and decreases activity of F1-F2 larvae (Blanc et al., 2020). Further, permethrin exposure is correlated with levels of lysophosphatidycholine, which is an important lipid for neurodevelopment.

#### 2.1.4 Social Stress

Social-ecological factors, such as race, disability, insurance status, family financial burden, residence in a metropolitan area, and exposure to violence, have been associated with ASD prevalence in communities (Brisendine 2017). These studies are an important addition to experiments examining toxicological and biological factors, given the unequal burden of social stress on families and communities, and the continuation of many social factors through several generations. Social stressor experiments in rats can mimic those experienced by humans, such as family conflict and depressed maternal care. Chronic social stress administered during F0 lactation impaired the maternal care of both F0 and F1 dams. Further, both male and female F2 offspring from this model showed decreased social behavior, as well as increased juvenile oxytocin (females), decreased adult prolactin (females), and decreased corticosterone (males and females) (Babb et al., 2014).

### 2.2 Transgenerational ASD Phenotypes and Gene Regulatory Changes

Building upon intergenerational findings, many animal studies have examined transgenerational phenotypes, or those that extend beyond the directly exposed generations. Several such studies have also reported gene regulatory changes such as differential DNA methylation, gene expression, and chromatin accessibility. Given epigenetic marks’ regulatory roles in gene expression (Jones 2012; Anastasiadi et al., 2018) and chromatin accessibility (Zhong et al., 2021), transcription level and chromatin changes may implicate DNA methylation as a potential mechanism conferring the effects of an environmental insult. While it is currently unclear whether changes in DNA methylation are causally related to multigenerational phenotypes, the studies discussed here show that increased investigation is worthwhile. Below are findings of studies that evaluate behavioral phenotypes as well as changes in DNA methylation, gene expression, and/or chromatin accessibility in at least three generations following a gestational exposure in an animal model.

#### 2.2.1 DNA Methylation

Synthetic glucocorticoids are a medication administered to pregnant people at risk of delivering preterm, and have been shown to affect offspring neurocognitive and behavioral function and alter the fetal epigenome (Crudo et al., 2013). When F0 pregnant guinea pigs were exposed to glucocorticoids, response to stress and stress-associated behaviors were altered in the F1-F3 generations through both maternal and paternal lineages, though the effects diminished through generations (Moisiadis et al., 2017). These behavioral changes correlated with changes in hippocampal gene expression and DNA methylation, particularly in RNApol II binding regions of small non-coding RNA genes (Constantinof et al., 2019). Methylation changes that correlated with gene expression changes were enriched for enhancer regions, while methylation changes at individual CpGs were enriched for promoter regions of small non-coding RNAs (Constantinof et al., 2019). Interestingly, the F3 generation showed the highest number of differentially expressed genes and differentially methylated regions between exposed versus non-exposed guinea pigs, followed by the F1 generation and lastly by the F2 generation (Constantinof et al., 2019). This indicates that transcriptional changes in the hippocampus may increase across generations following glucocorticoid exposure.

#### 2.2.2 Gene Expression

Valproic acid is a medication used to treat bipolar disorder and seizures and is a well-known risk factor for ASD in the offspring if taken while pregnant (Christensen et al., 2013; Wiggs et al., 2020). In mice studies of gestational valproic acid exposure, ASD-like phenotypes persisted through three generations following the exposure (Choi et al., 2016; Tartaglione et al., 2019). Transgenerational phenotypes included decreased sociability and increased marble burying (Choi et al., 2016), delayed righting reflex, increased motor activity, and reduced ultrasonic vocalizations (Tartaglione et al., 2019). Interestingly, the effects were stronger in the maternal lineage in Choi et al., but stronger in the paternal lineage in Tartaglione et al. Because different mouse strains were used in these studies (ICR versus CD-1), these differences may suggest a gene by environment interaction. Accompanying the altered phenotypes, mice in the valproic acid-exposed lineages showed increased expression of genes previously implicated in ASD pathology, such as excitatory postsynaptic proteins PSD-95 and Pax6 (Choi et al., 2016). Increased expression of endogenous retroviruses was also observed (Tartaglione et al., 2019), potentially because throughout history endogenous retroviruses have inserted into the human genome following infection (reviewed in Grandi and Tramontano 2018), which is a known risk factor for ASD if experienced during pregnancy (Lee et al., 2015; Zerbo et al., 2015).

Maternal infection during pregnancy activates the immune response and generates inflammation, a process that may alter central nervous system development and give rise to neurodevelopmental disorders (reviewed in Han et al., 2021; Brown et al., 2014). These effects have been suggested to be mediated by epigenetic factors (reviewed in Bergdolt and Dunaevsky 2019), and may also have implications for ASD-related behaviors past the F1 generation. Using a mouse model of prenatal immune activation with viral mimetic poly (I:C), Weber-Stadlbauer et al. showed reduced sociability and increased cued fear expression in three generations following exposure, *via* paternal lineage. Interestingly, the F2 and F3 offspring of immune-challenged ancestors showed increased behavioral despair, but this phenotype was not present in the F1 generation, again showing that novel phenotypes can appear multiple generations following exposure. Similarly, many genes were differentially expressed in the amygdalar complex of treatment versus control animals, with some changes being common across generations and some being generation-specific. 1,132 genes were differentially expressed in both F1 and F2 offspring, with enrichment for the DARPP-32 pathway, which has been previously associated with neuropsychiatric disorders (Kunii et al., 2014).

Bisphenol A (BPA) is an organic synthetic compound that exerts weak estrogenic activities and is associated with increased ASD risk (Stein et al., 2015; Hansen et al., 2021). ASD is diagnosed in males much more frequently than in females, at about a 4:1 ratio, and it has been posited that BPA exposure could play a role in ASD sex differences (Thongkorn et al., 2019). In mouse studies of gestational BPA exposure, delivered through chow, ASD-like phenotypes of reduced social recognition and activity persisted through three generations following the exposure (Wolstenholme et al., 2019). In F3 mice from the BPA lineage, expression levels of *Shank1* were significantly different from controls in both the hypothalamus and lateral septum at embryonic stage, day-of-birth, and juvenile stage. Interestingly, the direction of change was not consistent across time points (Wolstenholme et al., 2019). *Shank1* encodes a postsynaptic scaffolding protein, and mutations in this gene have been identified in individuals with ASD (reviewed in Monteiro and Feng 2017). In another study, differentially methylated regions were identified in the sperm of lineages of rats exposed to BPA, as well as DEET and TCDD (Manikkam et al., 2012), though behavioral phenotypes were not measured.

#### 2.2.3 Chromatin Accessibility

Anesthetics have also been associated with neurobehavioral abnormalities following prenatal exposure (reviewed in Andropoulos 2018), particularly if the exposure occurs early in pregnancy (Cui et al., 2021). In a transgenerational mouse study modeling human anesthesia, gestational exposure to sevoflurane led to anxiety and impairments in social interaction in the following three generations through the paternal lineage (Wang et al., 2021). While 38% of F1 showed these behavioral impairments, 44–47% of the F2 and F3 mice displayed them, again suggesting amplification across generations. To assess transcription factor distribution in the sperm genome of sevoflurane-treated versus control lines, the authors performed ATAC-sequencing, a method that analyzes chromatin accessibility using a Tn5 transposase that “tagments” open chromatin regions. ATAC-seq analysis of the F1 and F2 sperm showed 69 differentially accessible sites that are shared across the two generations of treated versus control lines. Differentially accessible sites in F1 sperm were enriched for genes involved in diseases of the nervous system and mental disorders and overlapped with ASD candidate genes such as *Arid1b*, *Ntrk2*, and *Stmn2* (Wang et al., 2021). The authors suggested that changes in chromatin accessibility and transcription factor binding may prevent DNA re-methylation during reprogramming of the epiblast, leading to downstream changes in gene expression (Kremsky and Corces 2020; Wang et al., 2021). This mechanistic possibility is explored further in the next section.

#### 2.2.4 Summary of Transgenerational Findings

Taken together, these studies show that DNA methylation, gene expression, and chromatin accessibility are often dysregulated in concert with altered behavioral phenotypes following a transgenerational exposure. Like the findings from intergenerational human studies, the maternal and paternal lineages in the transgenerational animal studies appeared to have different effects. Further, in several studies, the direction of change was not consistent across generations, and was often amplified in F3 compared to F1 and F2, indicating a potentially complex mechanism of transgenerational effects.

## 3 Potential Mechanisms for Multigenerational Risk for ASD

While multigenerational changes in phenotype as well as DNA methylation, gene expression, and chromatin accessibility following an environmental insult have been well documented, the mechanisms to explain such results are less well established. Current evidence for multigenerational epigenetic inheritance is reviewed below, as well as literature pointing towards a potential alternative mechanism.

### 3.1 Multigenerational Epigenetic Inheritance

The most studied epigenetic mechanism for disease risk being transferred through generations is the maintenance of DNA methylation at some chromosomal loci that escape the normal erasure and reestablishment in the germline; this is termed multigenerational epigenetic inheritance (MEI) (or transgenerational epigenetic inheritance (TEI) if the mechanism extends to non-exposed generations). Though there is significant evidence for MEI in plants (Hauser et al., 2011), yeast and nematodes (Fabrizio et al., 2019) and zebrafish (Cavalieri and Spinelli 2017), there is not currently substantial evidence for MEI in mammals.

MEI is often thought to be unlikely because DNA methylation patterns are generally not maintained through meiotic divisions. During development, DNA methylation is erased and reestablished through two genome-wide demethylation events: 1) after fertilization of the zygote and 2) during the formation of primordial germ cells, which are direct precursors to gametes (reviewed in Zeng and Chen 2019). These erasures allow for totipotency while reestablishment of DNA methylation marks commits cells to a fate. However, DNA methylation of the mouse, pig, and human germlines has been shown to exhibit incomplete erasure, (Kearns et al., 2000; Sutherland et al., 2000; Tang et al., 2015; Guo et al., 2017; Gómez-Redondo et al., 2021), with primordial germ cells retaining approximately 10% of their methylation marks (Seisenberger et al., 2012) while the inner cell mass of the blastocyst retains approximately 20% (L. Wang et al., 2014). This incomplete erasure indicates that some methylation marks are passed through the germline.

The most pronounced example of incomplete erasure occurs with imprinted genes, whereby DNA methylation marks prevent the expression of one parental allele, while the other parental allele is expressed. DNA methylation marks of imprinted genes are established in the germline of parents and retained through somatic cell divisions of the offspring. While some imprinted genes may have no biological relevance, others are necessary for proper development (reviewed in Tucci et al., 2019). Inappropriate imprinting may lead to neurodevelopmental disorders such as Angelman Syndrome and Prader-Willi Syndrome (reviewed in Butler 2020; Kalsner and Chamberlain 2015), which may also be caused by genetic defects such as uniparental disomy or chromosomal deletions.

Non-imprinted regions have also been shown to escape DNA methylation reprogramming (Tang et al., 2015). Mouse studies have shown that intracisternal A particles (IAPs) (Lane et al., 2003; Guibert et al., 2012), and LTR-ERV1 retroelements (Guibert et al., 2012) as well as other repetitive elements (Hajkova et al., 2002) escape reprogramming in primordial germ cells, while some retrotransposons escape reprogramming at the preimplantation embryo step (reviewed in (Zeng and Chen 2019). Interestingly, non-repetitive loci that escape reprogramming are enriched for genes involved in neurodevelopment and metabolism (Tang et al., 2015).

Regions may also experience MEI due to protection by bound transcription factors, as hypothesized by Kremsky and Corces 2020. One study found that 78% of CpGs maintain their methylation status following two rounds of reprogramming (Kremsky and Corces 2020). This faithful maintenance may be explained by transcription factors that bind CpG sites and prevent de-methylation and re-methylation, while unbound sites undergo reprogramming. Differential transcription factor binding may be explained by changes in chromatin accessibility (Wang et al., 2021). This possibility ties together the mechanisms of DNA methylation and chromatin accessibility to affect gene expression and potentially neurodevelopmental phenotypes.

In total, these studies raise the possibility of transmission of methylation marks at some loci through generations. This mechanism may help explain the ASD-related multigenerational phenotypes that follow environmental exposures, though further research is required.

### 3.2 Alternative Mechanism for Multigenerational Effects: Intermediate Phenotypes in F1

**FIGURE 2:**
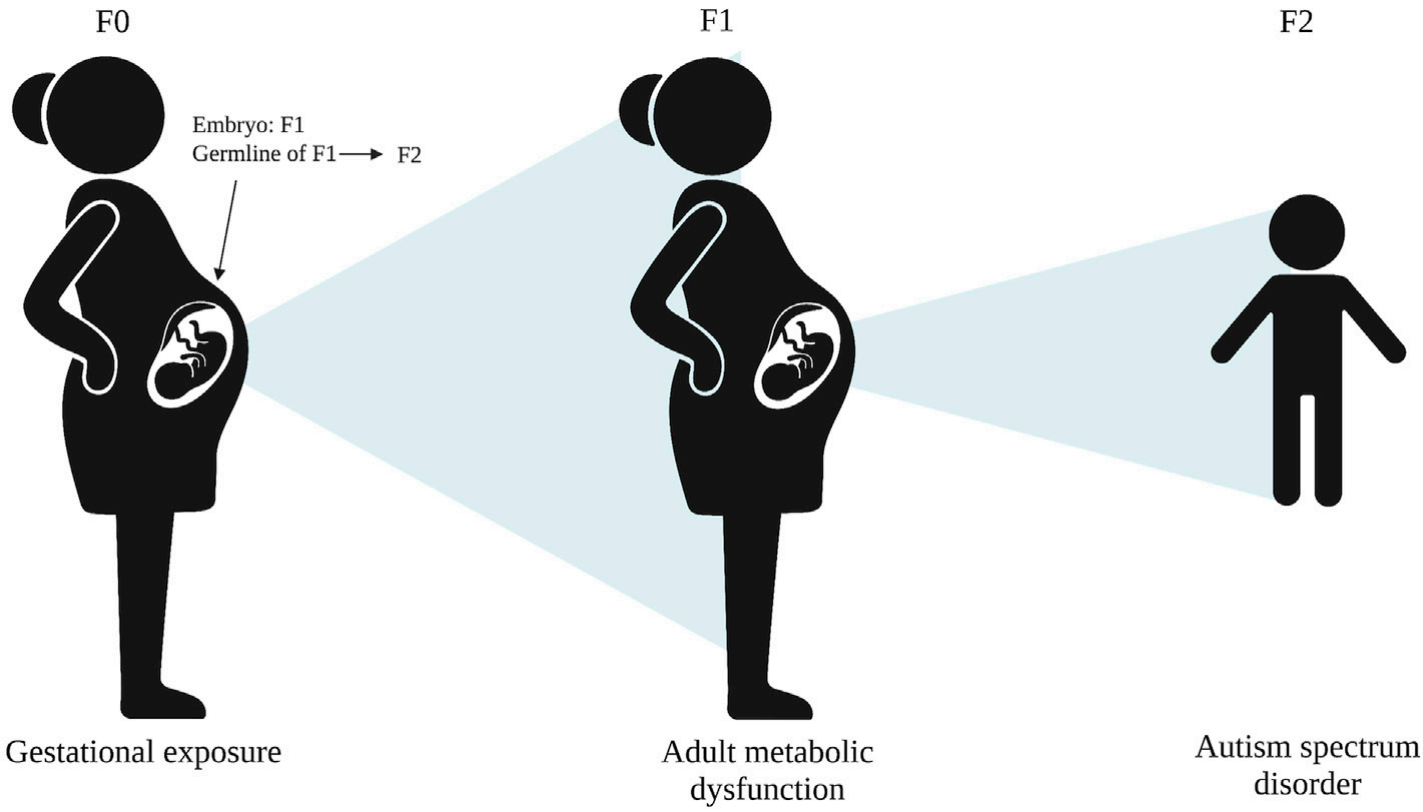
An alternative mechanism for adverse health effects, such as ASD in the F2 generation, whereby an intermediate phenotype, such as metabolic dysfunction, in F1 mediated the F0 exposure and F2 phenotype.

While MEI could be mechanistically involved with the transgenerational transmission of some phenotypes, alternative mechanisms are possible, particularly for intergenerational phenotypes. For example, an intermediate phenotype in the second generation (F1), such as metabolic dysfunction, could mediate the relationship between a gestational exposure in the first generation and adverse health effects, such as ASD, in the third generation or later (Figure 2).

Neurodevelopment and metabolism are closely linked, as the brain consumes ∼20% of the body’s calories, while only representing ∼2% of body weight. Many patients diagnosed with ASD or related disorders such as ADHD suffer from metabolic abnormalities including obesity (Mota et al., 2020; reviewed in; Hill et al., 2015). Metabolism is adaptive to environmental changes, such as nutrient availability and chemical exposures (reviewed in Koyama et al., 2020). In humans, exposure to chemicals such as dichlorodiphenyltrichloroethane (DDT) (La Merrill et al., 2020) and polychlorinated biphenyls (PCBs) (S.-L. Wang et al., 2008; Philibert et al., 2009; Langer et al., 2014) increases risk for metabolic disorders such as obesity and diabetes, and DNA methylation patterns and dysregulated gene pathways are predicted to mediate this risk (Ghosh et al., 2014). In turn, metabolic dysfunction, including obesity, during pregnancy has been associated with increased risk of ASD in the offspring (Li et al., 2016); this is again predicted to be mediated by altered DNA methylation (Lin et al., 2017). Additionally, regions that evaded genome-wide DNA demethylation in human primordial germ cells are enriched for genes that are expressed in the brain and are involved in neural development, as well as obesity-related phenotypes (Tang et al., 2015). These findings raise the possibility that metabolic dysfunction in the second generation could mediate environmental exposure in the first generation and increased risk for ASD in the third generation. This hypothesis, as well as other potential mechanisms, would benefit from studies specifically designed to evaluate their likelihood.

## 4 Gaps in the Research and Future Directions

The studies discussed in this review demonstrate that ASD and ASD-related phenotypes may occur in multiple generations following an environmental insult. Further, these phenotypes may be reflected by altered DNA methylation, as well as gene expression and chromatin accessibility, even if these epigenetic changes are not causal. While the papers discussed in this review yield important findings, it is important to note the limitations of multigenerational studies. Human studies often lack sufficient controls and numbers of participants, as well as access to biospecimens and metadata from multiple generations and diverse populations. Animal studies address many of the deficiencies of multigenerational human studies but have yet to show causality of specific epigenetic changes with altered phenotypes. Additionally, the difficulty of translating findings from animal studies to humans remains a challenge, particularly when examining diseases of complex etiology and clinical phenotypes, such as ASD. To help fill these critical gaps we have recently developed a systems biology approach that integrates multiple variables of exposures, genetics, demographics, and social determinants of health with regions of co-methylation (Mordaunt et al., 2022).

Despite the challenges faced by multigenerational investigations, the findings discussed in this review have important implications for the field of precision medicine, which seeks to integrate genetic, environmental, and lifestyle data to customize healthcare. Precision medicine is particularly relevant to ASD given the current paucity of diagnostic and treatment tools, as well as the numerous genetic and environmental risk factors (reviewed in Loth et al., 2016). In recent years, methods have been developed to utilize epigenetic information in clinical settings, enhancing the effectiveness of precision medicine (BLUEPRINT consortium 2016). However, the field could be further advanced by incorporating multigenerational epigenetic information and phenotypes. For instance, identifying markers of *in utero* and historical exposures at birth could help identify infants at risk for ASD, enabling early interventions that have been associated with improved outcomes (reviewed in Masi et al., 2017). Epigenetic biomarkers may also help identify sub-types of ASD as well as potential treatment options (Masi et al., 2017).

There remain many gaps in the mechanistic understanding of multigenerational phenotypes and DNA methylation. Further studies and improved methods are needed to elucidate precise mechanisms of action of environmental factors, gene-environment interactions, and multigenerational effects. The results of such studies may help to identify biomarkers and risk factors for disease, improving diagnostic and treatment practices as precision medicine develops.
